# Leptin in the Respiratory Tract: Is There a Role in SARS-CoV-2 Infection?

**DOI:** 10.3389/fphys.2021.776963

**Published:** 2021-12-22

**Authors:** Andreina Bruno, Giuliana Ferrante, Serena Di Vincenzo, Elisabetta Pace, Stefania La Grutta

**Affiliations:** ^1^Institute for Biomedical Research and Innovation, National Research Council, Palermo, Italy; ^2^Pediatric Division, Department of Surgical Sciences, Dentistry, Gynecology and Pediatrics, University of Verona, Verona, Italy

**Keywords:** immunity, leptin, microbiota, obesity, SARS-CoV-2

## Abstract

Leptin is a pleiotropic adipocytokine involved in several physiologic functions, with a known role in innate and adaptive immunity as well as in tissue homeostasis. Long- and short-isoforms of leptin receptors are widely expressed in many peripheral tissues and organs, such as the respiratory tract. Similar to leptin, microbiota affects the immune system and may interfere with lung health through the bidirectional crosstalk called the “gut-lung axis.” Obesity leads to impaired protective immunity and altered susceptibility to pulmonary infections, as those by severe acute respiratory syndrome coronavirus 2 (SARS-CoV-2). Although it is known that leptin and microbiota link metabolism and lung health, their role within the SARS-CoV2 coronavirus disease 2019 (COVID-19) deserves further investigations. This review aimed to summarize the available evidence about: (i) the role of leptin in immune modulation; (ii) the role of gut microbiota within the gut-lung axis in modulating leptin sensitivity; and (iii) the role of leptin in the pathophysiology of COVID-19.

## Introduction

**FIGURE 1:**
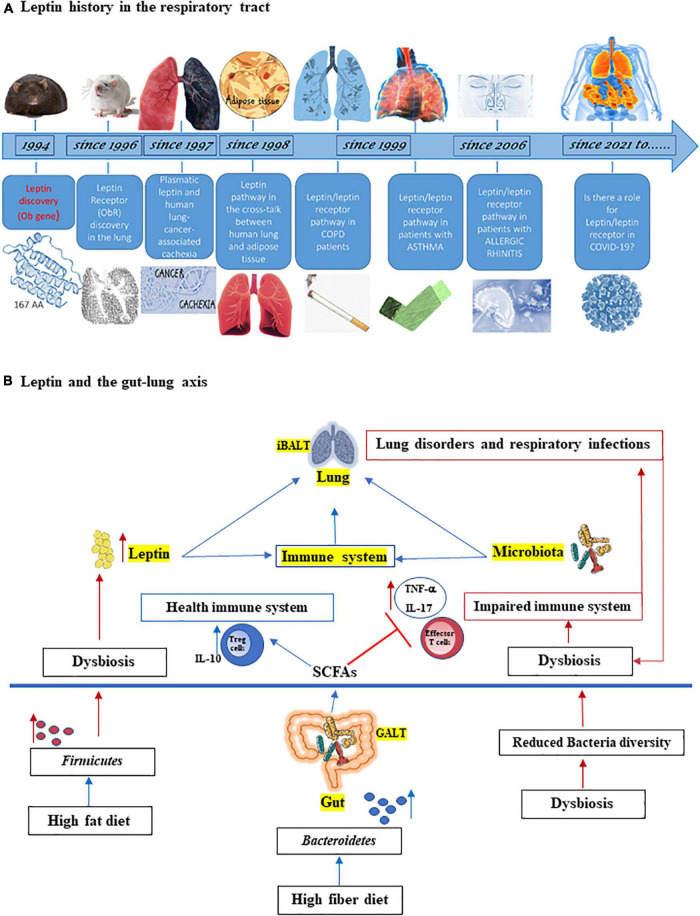
**(A)** Leptin history in the respiratory tract. Since its discovery 27 years ago, the adipocytokine leptin has provided a revolutionary framework for studying the physiological role of adipose tissue as an endocrine organ, also on respiratory tract. Leptin was discovered in mouse by Friedman group (Zhang et al., 1994) and soon leptin receptor (ObR) expression was found in the lung from rat (Wang et al., 1996). In 1997, it was studied as the correlation between serum leptin and lung cancer cachexia (Simons et al., 1997) and in 1998, the cross-talk between human lung and adipose tissue started to be identified (Kielar et al., 1998; Tankersley et al., 1998). Since 1999, leptin and its receptor are studied in patients with chronic obstructive pulmonary disease (COPD), asthma (Heuck and Wolthers, 1999; Takabatake et al., 1999; Bruno et al., 2005a,^2009^), and since 2006 also in patients with allergic rhinitis (Unal et al., 2006; Bruno et al., 2019). With coronavirus disease 2019 (COVID-19) pandemic, it has been hypothesized a role of leptin in severity disease (Guglielmi et al., 2021; Wang et al., 2021). **(B)** Leptin and the gut-lung axis. Both leptin and microbiota influence innate and adaptive immune system and are critical for maintaining homeostasis of the immune system in the lungs (iBALT = inducible bronchus-associated lymphoid tissue) and in the gut (GALT = gut-associated lymphoid tissue). High fiber diet can increase the prevalence of *Bacteroidetes* species as well as the production of short chain fatty acids (SCFAs), which maintain the health immune system through the induction of regulatory T cells (Tregs) and interleukin-10 (IL-10) production and by inhibiting inflammation. On the other hand, high fat diet can increase both adipose tissue and *Firmicutes* species, associated with dysbiosis, inflammation, and with increased of effector T cells and IL-17 and tumor necrosis factor-α (TNF-α) production as well as the level of circulating leptin. This latter is also increased by dysbiosis. In turn, lung disorders and respiratory infections boots dysbiosis.

Leptin adipocytokine is a pleiotropic hormone involved into widespread physiologic function, such as appetite and metabolic rate (Münzberg and Morrison, 2015; Mancuso et al., 2018), and in maintaining the homeostasis of immune system (La Cava and Matarese, 2004; Pérez-Pérez et al., 2017; Maurya et al., 2018; de Candia et al., 2021; Salum et al., 2021). The lung has been known as a sensitive and leptin-producing organ for more than 20 years with extensive research published for the role of leptin in the respiratory system, both in animals (Wang et al., 1996; De Matteis et al., 1998; Tsuchiya et al., 1999; Bergen et al., 2002) and humans (Bruno et al., 2005a,^2009^, ^2011^; Unal et al., 2006; Vernooy et al., 2009; Malli et al., 2010; Brandao-Rangel et al., 2021; Figure 1A).

High leptin concentrations are directly associated with obesity and/or the subsequent development of metabolic disease sequelae, such as insulin resistance, type 2 diabetes, and cardiovascular diseases (Ghadge and Khaire, 2019), all key risk factors associated with increased coronavirus disease 2019 (COVID-19) mortality (Kim et al., 2021; Mohammad et al., 2021). In addition, it has been assessed that an increased leptinemia is observed not only in patients with obesity and metabolic syndrome but also in patients who are not obese but affected by other inflammatory diseases, such as sepsis and respiratory infections (Vernooy et al., 2013; Birlutiu and Boicean, 2021; Karampela et al., 2021). Furthermore, increased value of leptin is one of the factors that raised the risk of non-alcoholic fatty liver disease presence in patients with prediabetes (Vesa et al., 2020) as well, after adjusting for body mass index (BMI) or fat mass, serum leptin levels result positively and independently associated with peripheral artery disease (Zahner et al., 2019).

Angiotensin converting enzyme 2 (ACE-2), a receptor required for the entry of severe acute respiratory syndrome coronavirus 2 (SARS-CoV-2) into the cells, is expressed on lungs, gut, pancreas, kidneys, heart, in vessels blood, and adipose tissue. Emerging evidence indicates that the ACE-2 expression is increased in individuals who are obese and overweight (Mohammad et al., 2021). Indeed, obesity appears to be a risk factor for worsening the severity of COVID-19 or of SARS-CoV-2 infection: large retrospective studies report a greater rates of obesity among patients with severe COVID-19 (Guerson-Gil et al., 2021; Zhou et al., 2021).

Similar to leptin, the gut microbiota is involved in the development and preservation of the immune system, energy homeostasis, and nutritional status (Belkaid and Hand, 2014; D’Argenio and Salvatore, 2015). Dysbiosis, altered microbiota composition, is associated with an increase in the proportion of bacteria with a pro-inflammatory profile, with a low-grade, persistent and systemic inflammation, and with poor outcomes in patients with COVID-19 (Magalhães et al., 2021; Moreira-Rosário et al., 2021). Interestingly, the gut microbiota may affect lung health through a crosstalk called the “gut-lung axis.”

The contribution of pro-inflammatory adipocytokine as leptin, together with the host microbiota, in modulating the immune system is a topic of interest in this research field.

## Leptin, at a Glance

Leptin is a 16-kD non-glycosylated hormone of 167 amino acids discovered in 1994 (Zhang et al., 1994) with a tertiary structure resembling that of members of the long-chain helical cytokine family. Leptin production is related to the amount of fat tissue (Considine et al., 1996), despite variability in plasma leptin concentration is independent from fat (Behnes et al., 2012) as leptin is produced by placenta, gastric mucosa, mammary gland, skeletal muscle, brain, intestine, bone marrow, and lymphoid tissues (Wolsk et al., 2012; Vernooy et al., 2013; Li et al., 2017; Pan et al., 2017; Pérez-Pérez et al., 2018).

Leptin receptor was found in lungs both from human and animals (Wang et al., 1996; De Matteis et al., 1998; Bruno et al., 2005a,^2009^) and in human inferior turbinates (Bruno et al., 2019). Since a while, it has been assessed that leptin displays many faces in the respiratory system, from lung embryogenic to maturation and to the control of ventilation (Tankersley et al., 1998; Takabatake et al., 1999; Jutant et al., 2021). All these functions are mainly related to the leptin-signaling pathway involvement in immune modulation (La Cava and Matarese, 2004; Procaccini et al., 2017).

Leptin modulates both innate and adaptive immune responses in monocytes/macrophages (Curat et al., 2004), neutrophils, eosinophils, effector, and regulatory T lymphocytes (Bruno et al., 2005b; Conus et al., 2005; Lourenço et al., 2016). Furthermore, a large amount of literature reports an important association between leptin and inflammation (Naylor and Petri, 2016; Becerril et al., 2018; Ziegler et al., 2019; Schoeman and Fielding, 2021).

Leptin modulation of inflammatory responses can directly contribute to the pathophysiology of COVID-19. Leptin activates monocytes promoting cytokine storm that contribute to severe respiratory distress syndrome and multiple-organ failure in COVID-19. Upon infection, patients with excessive fat mass would be prone to produce more leptin. High levels of leptin were associated with inflammatory mediators and disease severity in obese and not obese patients (Vernooy et al., 2013; Birlutiu and Boicean, 2021; Karampela et al., 2021; Wang et al., 2021). A state of low-grade, chronic inflammation, as observed in obesity, has been associated with altered leptin levels, impaired immune system, and host defenses: obese subjects are more susceptible to respiratory virus infection, to a greater severity of illness prolonged viral shed, increased viral diversity, and adverse endpoints after diseases, until death (Andersen et al., 2016; Aquino-Junior et al., 2018).

Leptin together with other cytokines, could be promising new biological markers and therapeutic targets in obesity-related diseases (Ghadge and Khaire, 2019; Bruno et al., 2021). As leptin deficiency/resistance has been associated with immune dysregulation and altered cytokine production, impairments of leptin signaling may hinder the cooperative interplay of the immunologic, metabolic, and neuro-endocrinologic processes (White et al., 2013; Guglielmi et al., 2021), potentially playing a role in driving the COVID-19 cytokine storm. In addition, leptin sensitivity may be modulated by gut microbiota (Heiss and Olofsson, 2018).

## The Microbiota as a Modulator of Leptin Sensitivity

The term “*microbiota*” identifies all microorganisms, mainly bacteria and a small number of fungi, archaea, and viruses, that live on the surface and inside our body (D’Argenio and Salvatore, 2015). Most of the adult human microbiota lives in the gut but it also colonizes the oral cavity, skin, vagina, and the lung (Dickson et al., 2016; Blum, 2017; Maschirow et al., 2019; Campisciano et al., 2021). These communities of microorganisms are essential for human physiology and survival and for this reason the microbiota is called the “forgotten organ” (O’Hara and Shanahan, 2006).

There is a strong link between the diet, the gut microbiota, and the effects on the metabolism of host (Tremaroli and Bäckhed, 2012). The gut microbiota regulates the host energy homeostasis as it is involved in the absorption of host nutrients, in maintaining the integrity of the intestinal immune barrier, in the regulation of host fat storage genes, and pathways that modulate appetite, intestinal motility, and energy expenditure (Backhed et al., 2004; Duca and Lam, 2014; Bagarolli et al., 2017). Alterations of the gut microbiota are associated with obesity and vice versa. Studies have shown that a high-fat diet can cause imbalances in the composition of the gut microbiota with a decrease of phylogenetic diversity and that germ-free animals are protected from this (Cani et al., 2008; Duncan et al., 2008; Bagarolli et al., 2017). At the same time, other studies have showed that the obese phenotype can be transferred by gut microbiota transplantation (Turnbaugh et al., 2006; Heiss and Olofsson, 2018).

Leptin sensitivity can be influenced by multiple factors, such as diet and gut homeostasis (Gabriel and Fantuzzi, 2019), which in turn is profoundly affected by gut microbiota asset. Microbiota metabolites, such as short-chain fatty acids (SCFAs) as acetate, propionate, and butyrate, can activate the specific signaling pathways in the host and regulate the secretion of hormones, such as glucagon-like peptide, peptide YY, and leptin itself, affecting the gut motility and the fat storage in the adipose tissue (Heiss and Olofsson, 2018). It was found that SCFAs, produced by the gut microbiota, stimulate the production of leptin in mouse adipocyte cultures through the activation of the G-protein coupled receptor (GPR) 41 and their oral administration in mice also increases the concentrations of circulating leptin (Xiong et al., 2004). In a study on diet-induced obese and type 2 diabetic mice, it has been assessed that prebiotics (good food for microbiota) improve leptin sensitivity in an altered gut microbiota composition, suggesting that gut microbiota modulations could be a novel therapeutic target to reset leptin sensitivity (Everard et al., 2011). Furthermore, it has been reported that leptin receptor deficient mice (db/db) have lower *Bacteroidetes* and higher *Firmicutes* proportions than wild-type mice (Rajala et al., 2014) as both leptin and adiponectin supplementation throughout the suckling period are able to modify both the intraepithelial lymphocytes and gut microbiota composition in mice (Grases-Pintó et al., 2019). An experimental study performed in humans in overweight/obese mothers, with high leptin concentration in their breast milk, reported a lower abundance of *Proteobacteria* phylum in the infant gut microbiota (Lemas et al., 2016). Specific bacterial strains are associated with the release of leptin. A study demonstrates that amounts of *Bifidobacterium* and *Lactobacillus* correlate positively with plasma concentrations of leptin (Queipo-Ortuno et al., 2013). Intestinal dysbiosis has been reported to be associated with chronically increased leptin levels and decreased sensitivity to leptin, through the induction of the suppressor of cytokine signaling 3 (SOCS3) and the suppression of the brain-derived neurotrophic factor (BDNF) expression in the hypothalamus (Schéle et al., 2013), and decreased expression of obesity-suppressing neuropeptides in the central nervous system (Yao et al., 2020). All evidence aimed to assess a possible modulation by microbiota on leptin expression/sensitivity and vice versa.

Gut microbiota, as leptin, influences and modulates inflammation and immune systems (Macpherson and Harris, 2004; Lynch and Pedersen, 2016; Bizzoca et al., 2020; Mohammadi et al., 2020; Yang et al., 2020). Commensal bacteria are recognized by the innate immune system and thus the microbiota plays a role in regulating the development, homeostasis, and function of innate and adaptive immune cells (Brestoff and Artis, 2013), avoids inflammation and bacterial translocation and hinders the colonization of pathogens (Maschirow et al., 2019).

In addition, gut microbiota dysbiosis is associated with lung disorders and respiratory infections (Trompette et al., 2014; Shukla et al., 2017). Changes in species and the proportion of bacteria in the gut are associated with asthma, lung disease, and allergic inflammation (Kalliomaki et al., 2001; Russell et al., 2013). Conversely, changes in the lung microbial community due to viral infections modify the composition of the gut microbiota leading to intestinal dysbiosis (Ichinohe et al., 2011). All these findings confirm that gut microbiota systematically influences the lung microbiota and this strictly interconnection is termed “gut-lung axis” (Marsland and Gollwitzer, 2014; McAleer and Kolls, 2018; Dang and Marsland, 2019).

## Leptin and Gut-Lung Axis

A balance between host and gut microbiota is crucial to keep a healthy intestinal barrier and for healthy metabolism. Microbiota is believed to contribute to metabolism in humans as it has been reported that differences in the composition of the microbiota are related to obese or lean individuals (Turnbaugh et al., 2009). Moreover, microbiota plays a fundamental role in optimal immune homeostasis (Wu and Wu, 2012).

Gut-lung axis communicates through a bi-directional pathway in which endotoxins, or microbial metabolites, may affect the lung through the blood and, conversely, the lung inflammation affects the gut microbiota. To further support this axis in pathological contexts, it has been demonstrated a link between bowel and lung inflammatory diseases (Wypych et al., 2019; Raftery et al., 2020). This axis is established because there can be a direct seeding of intestinal bacteria into the lung through reflux and aspiration, but also because some products of the intestinal bacterial metabolism and nutrition can influence the composition and functionality of the lung microbiota. The production of SCFAs from dietary fiber by the intestinal microbiota increases the presence in the lung of dendritic cells with high phagocytic capacity and reduces the ability to promote the effector function of Th2 cells, thereby improving the allergic airway inflammation (Trompette et al., 2014). Multiple mechanisms could be postulated to explain how gut microbiota modulates lung immune responses. In this regard, it has been shown that human lung tissues expressed SCFAs receptor, free fatty acids receptor 2 and 3 (FFAR2 and FFAR3) (Liu et al., 2021), and the activation of these receptors influences the expression interleukin-1β (IL-1β) and in turn lung immune tone (Mizuta et al., 2020) and airway hyperactivity. Some micronutrients exert a relevant effect on gut microbiota leading to the production of specific metabolites that affect immune systems and in turn chronic disease development or evolution (Espírito Santo et al., 2021). High fiber intake can limit emphysema progression and mitigates the inflammatory response in cigarette smoke-exposed emphysema mice (Jang et al., 2021). A systematic review (Gabriel and Fantuzzi, 2019) analyses the relationship between SCFAs and leptin metabolism: it concludes that body fat, rather than SCFAs, remains the main driver for leptin synthesis *in vivo* and that the activation of FFAR3 increases leptin release and expression *in vitro*.

Furthermore, a cross-sectional study design (Yang et al., 2017) reports that the gut microbiota is associated with cardiorespiratory fitness in women, regardless of age and dietary intakes, with increased *Eubacterium rectale-Clostridium coccoides (EreC*) *and Enterobacteria* but lower *Bacteroides* and with low aerobic fitness and low maximum oxygen uptake (VO2 max). While VO2 max is negatively correlated with fat percentage and leptin, *EreC* is positively associated with fat percentage and leptin, but the relationship between VO2 max and *EreC* is confused by body fatness as the observed differences disappeared after adjusting of the fat percentage.

Recent evidence supports a relevant role of gut-lung axis in acute respiratory distress syndrome (ARDS) (Dickson et al., 2016), COVID-19 (Allali et al., 2021), and chronic obstructive pulmonary disease (COPD) pathogenesis (Lai et al., 2021). Several current studies are elucidating the mechanisms of how microbiota regulate lung inflammation and are providing useful information for considering the use of probiotic, prebiotic, and postbiotic therapies for lung disease, such as COVID-19 (Tsai et al., 2019; Gasmi et al., 2021). Microbial-derived components (postbiotics) elicit the activation of downstream cascades capable to modulate both local and systemic immune responses.

## Leptin, Respiratory Health, and Sars-CoV-2 Infection

According to increasing scientific evidence, leptin can modulate respiratory health through pleiotropic actions (Jutant et al., 2021). First, leptin has been reported to play a role in lung development and in the maturation of fetal lungs, as it seems to be involved in surfactant proteins production by fetal type II cells (Torday et al., 2002). In addition, leptin can modulate bronchial diameter, by counteracting the parasympathetic effect on the airways (Arteaga-Solis et al., 2013). Finally, congenital leptin-deficient patients show defects in immunity and are at risk of death due to infections (Díez et al., 2008). Dysregulated leptin production and activity could be involved in the pathogenesis of several pulmonary diseases, such as COPD, idiopathic pulmonary fibrosis, lung cancer, and pulmonary arterial hypertension. Interestingly, its role appears to be both protective through bronchodilation and negative by promoting inflammation in patients with asthma. Furthermore, leptin appears to be protective against respiratory infections. In patients hospitalized for pneumonia, leptin levels were inversely correlated with markers of inflammation (Jutant et al., 2021). More recently, a significant association between high plasma leptin levels and risk of severe respiratory infections was found in a cohort of ambulatory patients, independent on BMI and other risk factors (Ubags et al., 2016). In obese patients with a viral infection, altered leptin sensitivity may contribute to a dramatic pro-inflammatory cytokine response and to an inefficient response to infection (Alti et al., 2018). It has been suggested that leptin could be involved in the etiology of several effects commonly observed in patients with COVID-19. For instance, the frequently reported anosmia (Gane et al., 2020) may be partly ascribed to the ability of leptin to alter the olfactory epithelium (Savigner et al., 2009). One study recently reported a positive correlation between serum leptin levels and BMI of adult patients infected with SARS-CoV-2 (van der Voort et al., 2020). Wang et al. observed that patients with COVID-19 with a high BMI had significantly high levels of leptin, which were associated with inflammatory mediators and disease severity in such patients. Of note, leptin levels are increased in patients with COVID-19 compared with controls as well as in severe patients with COVID-19 compared with mild patients (Wang et al., 2021). Therefore, it seems that, upon infection, patients with excessive fat mass are prone to produce more leptin, which in turn activates monocytes promoting that cytokine storm that has been recognized to contribute to severe respiratory distress syndrome and multiple-organ failure in COVID-19. Furthermore, leptin is inhibited by ACE-2 *via* alamandine production and activation of the MrgD-receptor/c/Src/p38MAPK pathway (Uchiyama et al., 2017). Therefore, it has been hypothesized that in obese patients infected by SARS-CoV-2, the impaired ACE-2 function after the viral binding may increase leptin levels. This may contribute to the hyperinflammatory pulmonary response frequently observed in obese patients infected with SARS-CoV-2 (Guglielmi et al., 2021).

In summary, a weakened immune response can end in a heightened cytokine release that can prove fatal. However, the role of leptin in the pathogenesis of SARS-CoV-2 needs further investigation to be fully clarified.

## Discussion and Conclusion

The COVID-19 pandemic continues to represent the worst health threat worldwide and to cause morbidity and mortality with more than 5,000,000 death cases reported to the WHO by November 2021.^1^

It has been assessed that the healthy microbiota of upper and lower respiratory tract plays several important roles in the development and maintenance of respiratory tract and whole organism homeostasis and the viral infections, such as that caused by SARS-CoV-2 may perpetuate a systemic inflammation *via* gut-lung axis (Banerjee et al., 2020; Belanger et al., 2020; Gheblawi et al., 2020). Indeed, gut dysbiosis may be linked to the onset of several pulmonary diseases, such as asthma (Huang et al., 2021.), COPD (Raftery et al., 2020), cystic fibrosis (Thavamani et al., 2021), and lung infections (Bajinka et al., 2021). A connection between the lungs and gut has been widely demonstrated in both human and mouse studies. Inducible bronchus-associated lymphoid tissue (iBALT) and gut-associated lymphoid tissue (GALT) are strictly interconnected and both leptin and microbiota are important factors responsible for interactions between these two sites (Figure 1B).

Leptin could represent an important player in the gut-lung axis (Di Renzo et al., 2020). It has been hypothesized that in obese patients infected by SARS-CoV-2, the impaired ACE-2 function after the viral binding may increase leptin levels, thereby contributing to the hyperinflammatory pulmonary response (Guglielmi et al., 2021). This framework may explain the occurrence of respiratory failure which has been commonly observed in overweight/obese patients. It has been widely assessed that obesity has an adverse effect on respiratory physiology both for mechanical factors and for impaired adipocyte-mediated immune function by increased levels of pro-inflammatory cytokines and by decreased anti-inflammatory adipokines. Majority of the observational and retrospective cohort studies on thousands of patients with COVID-19 report that obese subjects are at increased risk of severe disease and increased mortality due to COVID-19 (Asare et al., 2020; Gazzaruso et al., 2020; Pettit et al., 2020; Guerson-Gil et al., 2021; Rapp et al., 2021). Since respiratory failure usually takes place at 8–12 days from the initial signs of infection, there would be a window of opportunity to intervene, for instance by downregulating the leptin production (van der Voort et al., 2020). A relevant aspect also concerns the role of the leptin-immune axis, as leptin can impair antibodies production and class switching of immunoglobulin. Indeed, increased leptin levels are recognized as the mediating factor linking metabolism and immunity and are thought to predispose to increased morbidity and mortality for SARS-CoV-2 infection through an impairment of the immune response (Rebello et al., 2020).

On the basis of the evidence here provided, it could be possible to hypothesize a relevant role of leptin in the increased levels of pro-inflammatory mediators in the obese patients with COVID-19. Leptin can disrupt the release of anti-inflammatory cytokines and anti-inflammatory adipokines, leading to the impairment of the normal immune function and perpetuating the progression and the severity of chronic diseases as well as infections, such as COVID-19. However, nowadays, it is still to be explored whether leptin could be used in clinical practice as a pro-inflammatory biomarker of disease progression and severity to predict the patient prognosis (Wang et al., 2021).

At the same time, the interest of the scientific pre-clinical and clinical research in gut microbiota is growing, but the experimental studies in this field are still at the beginning. Our review is illustrative and aim to focus the next-future research for lung diseases and COVID-19 specifically in the field of the interaction between leptin and microbiota, as both widely involved in the regulation and immune and inflammatory systems. Anyway, the presence of the functional leptin receptor in the lung together with evidence of local leptin production, supports the concept that leptin plays an important role in lung health (Vernooy et al., 2013).

**FIGURE 2:**
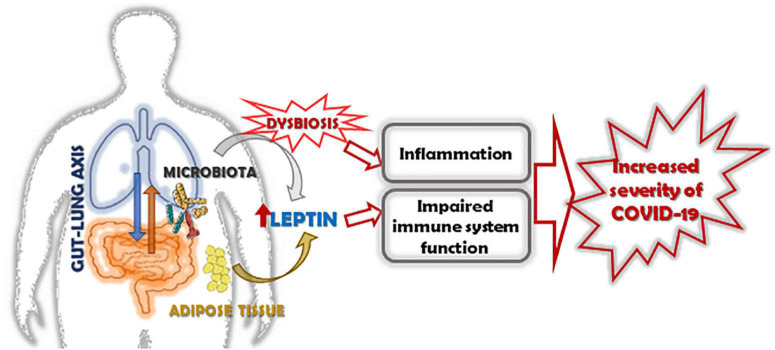
Role of leptin in increasing the severity of COVID-19 in obese subjects. The altered expression of leptin/leptin receptor pathway and the increase of leptin resistance in obese subjects, together with the alteration of microbiota, induces inflammation, and impairs the function of the immune system. In this context, leptin and dysbiosis could be key factors associated with increased severity of COVID-19 in obese subjects.

In conclusion, obesity-associate chronic inflammation impairs immune function and increases ACE-2 expression resulting in an increased disease severity and worse clinical outcome in obese subjects with COVID-19. The goal in this field is to understand the contribution of pro-inflammatory adipocytokine as leptin, together with the host microbiota in modulating the immune system. We strongly suggest that the next prospective studies in lung infections may be integrated and be given an interdisciplinary approach, included nutritional status, and gut microbiota, as these new insights could be translated into preventive and therapeutic measures for COVID-19 (Figure 2).

## Author Contributions

AB and SD: conceptualization. AB, EP, SD, and GF: writing original draft. AB, EP, GF, and SL: review and editing. All authors contributed to the article and approved the submitted version.

## Conflict of Interest

The authors declare that the research was conducted in the absence of any commercial or financial relationships that could be construed as a potential conflict of interest.

## Publisher’s Note

All claims expressed in this article are solely those of the authors and do not necessarily represent those of their affiliated organizations, or those of the publisher, the editors and the reviewers. Any product that may be evaluated in this article, or claim that may be made by its manufacturer, is not guaranteed or endorsed by the publisher.

## Footnotes

1
https://covid19.who.int
